# Multimodal inhibitory effect of matcha on *Porphyromonas gingivalis*

**DOI:** 10.1128/spectrum.03426-23

**Published:** 2024-05-21

**Authors:** Ryoma Nakao, Ayami Takatsuka, Kengo Mandokoro, Naoki Narisawa, Tsuyoshi Ikeda, Hideki Takai, Yorimasa Ogata

**Affiliations:** 1 Department of Bacteriology I, National Institute of Infectious Diseases, Tokyo, Japan; 2 Department of Food Bioscience and Biotechnology, College of Bioresource Science, Nihon University, Kanagawa, Japan; 3 Department of Pharmaceutical Sciences, Sojo University, Kumamoto, Japan; 4 Department of Periodontology, Nihon University School of Dentistry at Matsudo, Chiba, Japan; Institute of Hydrobiology Chinese Academy of Sciences, Wuhan, China

**Keywords:** periodontal disease/periodontitis, *Porphyromonas gingivalis*, *Camellia sinensis*, matcha, membrane fluidity, membrane potential, catechins, FimA, mouthwash, probing pocket depth

## Abstract

*Porphyromonas gingivalis* has been associated with progression of periodontitis, characterized by inflammation and destruction of periodontal tissues. Here, we report that matcha, a product of *Camellia sinensis*, hampers the adherence and survival of *P. gingivalis* through multiple tactics. Matcha extract (ME) inhibited the growth not only of *P. gingivalis* but also of *Prevotella nigrescen*s and *Fusobacterium nucleatum*, while it did not inhibit growth of nine species of oral streptococci and *Aggregatibacter actinomycetemcomitans*. ME-mediated *P. gingivalis* growth inhibition was characterized by both morphological and physiological changes at the bacterial envelope, which were accompanied by nano-particle formation and decreased membrane fluidity/permeability without loss of membrane integrity. ME also triggered autoaggregation of *P. gingivalis* in a major fimbriae (FimA)-dependent manner. In addition, adherence of *P. gingivalis* was dramatically inhibited by ME, irrespective of fimbriae. Furthermore, a structure-activity relationship study tested a series of catechins isolated from ME and identified the pyrogallol-type B-ring of catechins as essential for *P. gingivalis* growth inhibition. In a clinical study to assess the microbiological and therapeutic effects of matcha mouthwash in patients with periodontitis, the *P. gingivalis* number in saliva was significantly reduced by matcha mouthwash compared to the pre-intervention level. A tendency toward improvement in probing pocket depth was observed in the matcha group, although the difference was not statistically significant. Taken together, we present a proof of concept, based on the multimodal inhibitory effect of matcha against *P. gingivalis*, and that matcha may have clinical applicability for prevention and treatment of periodontitis.

**IMPORTANCE:**

Periodontitis, a multifactorial inflammatory disease of the oral cavity, results in alveolar bone destruction, and is a major cause of tooth loss of humans. In addition, emerging evidence has demonstrated associations between periodontitis and a wide range of other chronic inflammation-driven disorders, including diabetes mellitus, preterm birth, cardiovascular disease, aspiration pneumonia, rheumatoid arthritis, cognitive disorder, and cancer. In the present study, we report that matcha, a product of *Camellia sinensis*, hampers *Porphyromonas gingivalis*, a major periodontal pathobiont, in not only a series of *in vitro* experiments but also a pilot intervention clinical trial of patients with periodontitis, in which matcha mouthwash statistically significantly reduced the *P. gingivalis* number in saliva, as compared to the pre-intervention level. Taken together, we suggest that matcha may have clinical applicability for prevention and treatment of periodontitis.

## INTRODUCTION

Oral health is an indispensable part of overall general health and well-being. Periodontal disease is an infection-driven chronic inflammatory disease that affects the tissues supporting the teeth. It has been reported that approximately 90% of adults have periodontal diseases to varying degrees, i.e.*,* both gingivitis and periodontitis, according to the global database of oral health, The WHO Oral Health Country/Area Profile Programme (CAPP) (1). In a study of the global burden of oral health conducted from 1990 to 2017, the age-standardized prevalence of severe periodontitis was 9.8%, representing 0.8 billion cases worldwide (2). Current dental practice strongly recommends a shift from the conventional curative approach toward a preventive approach, which includes promotion of oral health within families, schools, and workplaces, as well as within the primary health care system.

*Porphyromonas gingivalis*, a Gram-negative asaccharolytic oral bacterium, has been considered an important player in periodontal biofilms, which are involved in initiation and progression of periodontal diseases (3–5). Recent studies showed that *P. gingivalis* induced oral dysbiosis with host immune subversion in animal models (6, 7). Although the population of *P. gingivalis* within the periodontal pockets microbiota is small, this keystone bacterium-specific infection can change a normally benign microbiota into a dysbiotic one (8). Thus, elimination of *P. gingivalis* in the oral cavity has been at the center of attention for more than three decades in periodontology.

*Camellia sinensis* is a natural product well-studied for its antimicrobial activities against a wide range of pathogens including pathogenic *Escherichia coli* (9) and severe acute respiratory syndrome coronavirus 2 (10). Regarding the anti-*P*. *gingivalis* activity of the tea plant, several reports showed inhibitory effects on *P. gingivalis* growth and adherence to human epithelial cells (11–13). An epidemiological study of 940 Japanese men showed an inverse correlation between the intake of green tea and clinical parameters, including mean probing pocket depth and bleeding on probing (BOP) by an unknown mechanism (14), although as a cross-sectional study, a clear cause-and-effect relationship was lacking. Regarding biologically active compounds of *C. sinensis*, epigallocatechin-3-*O*-gallate (EGCG) was found to exhibit antimicrobial activity or inhibit biofilm formation of *P. gingivalis* in several reports (15, 16), although the mechanism behind anti-*P*. *gingivalis* action by *C. sinensis* is not fully understood in terms of the dynamics of membrane biology and physiology.

Matcha, one of the products made from *C. sinensis*, has been used not only for Japanese traditional ceremonies, but is a globally popular ingredient also used in confectionery sweets and beverages. Matcha is made from the raw leaves of the tea plant grown under special culture conditions in the absence of most sunlight. Due to this culturing method, the chemical composition of matcha is different from that of other tea products made from plants of the same botanical origin, such as black tea and green tea (17).

There is little information on the antibacterial effect of matcha on *P. gingivalis*. In addition, the clinical implications of topical matcha application for periodontal diseases have not yet been investigated. Here, we report that matcha extract (ME) hampered *P. gingivalis* biology at different life stages through multiple mechanisms. ME interfered with the *P. gingivalis* cell membrane, significantly affecting its morphology and physiological functions. In addition, our mechanistic study also revealed involvement of FimA fimbriae in ME-dependent autoaggregation. In an intervention study of patients with periodontitis, the *P. gingivalis* number in saliva was significantly reduced by matcha mouthwash. A tendency toward improvement in probing pocket depth (PPD) was also detected in the matcha mouthwash group. We discuss the clinical applicability of matcha for prevention and treatment of periodontitis on the basis of its multimodal inhibitory effect against *P. gingivalis*.

## MATERIALS AND METHODS

### ME and ME-derived compounds

Matcha was prepared from whole leaves of the tea plant harvested in the regions of Nara, Kyoto, Mie, and Shiga prefectures in Japan, situated from 33°43´ to 35°46´ N latitude and 134°51´ to 136°59´ E longitude. Component analysis of three lots of matcha and two lots of ordinary Japanese green tea was carried out by the Japan Food Research Laboratories (Tokyo, Japan), summarized in Fig. S4. Firstly, in order to identify a suitable solvent that would yield matcha extract containing biologically active components, the matcha was subjected to four different solvents as follows: (i) mixed solution of *n*-hexane:acetone:ethanol:toluene at the ratio of 10:7:6:7; (ii) ethanol; (iii) 1% formic acid in H_2_O solution; and (iv) H_2_O-acetone (1:1) solution. All these extracts were obtained as the supernatant after ultrasonicating for 4 h. Then, the supernatant was air dried, measured as dry weight, and redissolved in dimethyl sulfoxide (DMSO) to yield each extract at the concentration of 10 mg/mL. The H_2_O-acetone extract showed the strongest activity among four different extracts in terms of *P. gingivalis* growth inhibition as well as auto-aggregative action (data not shown). The H_2_O-acetone (1:1) extract was named ME, and used for all *in vitro* assays and for compound isolation. The work flow to isolate ME-derived compounds is described in Fig. S2.

### Bacterial strains and growth conditions

All strains used in this study are shown in Table 1. Three strains of *P. gingivalis* and one strain of *Prevotella nigrescens* were grown in brain heart infusion (BHI) broth supplemented with hemin (5 µg/mL) and menadione (1 µg/mL) (HM) (18) or on BHI blood agar plates (BAP) containing HM.(18) *P. gingivalis* strain ATCC 33277 (19) was mainly used to examine the effect of ME in detail. In part of the present study, we also used the following five isogenic ATCC 33277 derivatives: *fimA*^−^ (a major fimbriae FimA mutant), *mfa1*^−^ (a minor fimbriae Mfa1 mutant), *fimA^−^ mfa*1^−^ (a fimbriae double mutant lacking FimA and Mfa1) (20), *rgpA*^−^ *rgpB^−^ kgp^−^* (a triple gingipain mutant lacking RgpA, RgpB, and Kgp) (21), and *porK^−^* (a type IX secretion system [T9SS] mutant lacking an outer membrane lipoprotein PorK) (22). BHI broth and BHI BAP (without HM) were used to maintain strains of the other oral bacteria, including streptococci, *Aggregatibacter actinomycetemcomitans*, and *Fusobacterium nucleatum*. All oral bacteria were grown in an anaerobic chamber (miniMACS anaerobic workstation; Don Whitley Scientific Ltd., Shipley, UK) in 80% N_2_, 10% H_2_, and 10% CO_2_ at 37°C. A laboratory *Escherichia coli* strain BW25113, which was used as a model strain for bacterial membrane potential assay, was maintained in Luria-Bertani (LB) broth and on LB agar under aerobic conditions.

**TABLE 1:** MIC (μg/mL) of ME against a wide range of oral bacteria

Group^*a*^	Strain	MIC (µg/mL)
Periodontopathic bacteria (red)	*Porphyromonas gingivalis* ATCC 33277	250
Periodontopathic bacteria (red)	*P. gingivalis* W83	125
Periodontopathic bacteria (red)	*P. gingivalis* W50	250
Periodontopathic bacteria (orange)	*Prevotella nigrescens* ATCC 33563	250
Periodontopathic bacteria (orange)	*Fusobacterium nucleatum* no. 20	250
Periodontopathic bacteria (NA)	*Aggregatibacter actinomycetemcomitans* Y4 (serotype b)	>500
Periodontopathic bacteria (NA)	*A. actinomycetemcomitans* ATCC 29522 (serotype b)	>500
Oral commensal bacteria (yellow)	*Streptococcus anginosus* ATCC 33397	>500
Oral commensal bacteria (yellow)	*Streptococcus cristatus* ATCC 51100	>500
Oral commensal bacteria (yellow)	*Streptococcus gordonii* ATCC 10558	>500
Oral commensal bacteria (yellow)	*Streptococcus mitis* ATCC 6245	>500
Oral commensal bacteria (yellow)	*Streptococcus mutans* UA159	>500
Oral commensal bacteria (yellow)	*Streptococcus oralis* no.10	>500
Oral commensal bacteria (yellow)	*Streptococcus salivarius* ATCC 9759	>500
Oral commensal bacteria (yellow)	*Streptococcus sanguinis* ATCC 10556	>500
Oral commensal bacteria (yellow)	*Streptococcus sobrinus* ATCC 6715	>500

^*a*^ According to classification based on DNA-DNA hybridization with subgingival samples of patients with periodontitis and healthy subjects (4), oral bacterial species were categorized into three microbial complexes (yellow, purple, and green), which are described in parentheses in the “Group” column. Whereas most *Streptococcus* species are representative of species of the yellow complex, *P. gingivalis* is representative of species of the red complex. The red complex is primarily found in deeper periodontal pockets and its presence is usually preceded by species of the more diverse orange complex. In contrast, those of the yellow complex are considered to be generally associated with healthy sites. MIC, minimum inhibitory concentration; NA, not allocated to any complex.

### Growth inhibition assay

Growth assays were performed to examine the inhibitory effect of ME or ME-derived compounds on the bacterial suspension at the concentration of 1 × 10^8^ colony-forming units (CFU)/mL in BHI-HM broth or BHI broth in a 96-well flat-bottom polystyrene microtiter plate (3595, Coring, New York, NY, USA). For minimum inhibitory concentration (MIC) determination, each strain was subjected to the samples prepared by microdilution methods according to the Clinical and Laboratory Standards Institute protocols (23, 24), with some modifications. Growth was monitored as turbidity at *A*_620_ of the bacterial suspension in a 96-well plate at different time points using a micro-plate reader (Cytation 5, BioTek, Winooski, VT, USA). The broth contained a twofold dilution series of matcha extracts or matcha-derived compounds prepared at final concentrations ranging from 31.25 to 500 µg/mL or 62.5 to 500 µM, respectively.

### Killing assay to assess bactericidal activity

The bactericidal activity of ME was evaluated using a killing assay. Aliquots of *P. gingivalis* cell suspension standardized at 1 × 10^4^ CFU/mL were exposed to ME at different concentrations or DMSO (vehicle control). The cell suspensions were inoculated onto BHI-HM BAPs in duplicate at different time points, and cultured at 37°C under anaerobic conditions for at least 10 days. Killing activity was defined as [CFU (tested sample)/CFU (baseline, untreated sample)] × 100 (%).

### Field emission scanning electron microscopy (FE-SEM)

*P. gingivalis* cells treated with ME for 30 min was washed with phosphate-buffered saline, pH 7.4 (PBS), and then added to poly-L-lysine-coated cover slips for 10 min. Fixation of attached bacteria with 2.5% glutaraldehyde and 2% paraformaldehyde for 30 min was followed by three washes with PBS. After ethanol dehydration, the samples were immersed in isoamyl acetate, then dried by critical point drying and coated with osmium vapor using an osmium plasma coater, and subjected to FE-SEM (Regulus8220 or Regulus8600, Hitachi High-Tech, Tokyo, Japan).

### Transmission electron microscopy (TEM)

For TEM analysis, *P. gingivalis* cells were allowed to adhere to carbon-coated grids for 1 min at room temperature (15°C–24°C), and then negatively stained with 2% uranyl acetate. TEM analysis was performed using an H-7700 (Hitachi High-Tech).

### High-speed atomic force microscopy (HS-AFM)

Morphological change of *P. gingivalis* cells treated with ME was monitored by a HS-AFM (BIXAM, Olympus Corp., Tokyo, Japan), as described previously (25, 26). Briefly, *P. gingivalis* ATCC 33277 cells standardized at 1 × 10^9^ CFU/mL with PBS were incubated for 5 min to enhance the immobilization on glass slides (SF17370, Matsunami Glass, Osaka, Japan). ME was administrated to attached *P. gingivalis* cells using a winged needle (SL-23CK, Terumo, Tokyo, Japan) connected to a glass micro-syringe (250 µL volume, 708-SNR, Hamilton, Reno, NV, USA). After injection, the spatiotemporal transition of the bacterial surface was continuously captured by a high-speed 3D scanner. Commercially available cantilevers BL-AC10DS-A2 (Olympus Co., Ltd.) and USC-F0.8-k0.1 (Nanoworld AG, Neuchâtel, Switzerland) were used for the high-speed tip scanning. The area of *P. gingivalis* cells every 30 s after treatment with 1% DMSO (vehicle control) or ME was measured using HS-AFM images with the Fiji image processing package.

### Membrane fluidity assay

Membrane fluidity of *P. gingivalis* cells was measured by spectroscopic analysis with a fluorescent membrane dye, laurdan (6-dodecanoyl-N,N-dimethyl-2-naphthylamine) (27, 28), with some modifications. The principle of this assay depends on the character of laurdan, which intercalates into the membrane bilayer and displays an emission wavelength shift depending on the amount of water molecules in the membrane. In brief, *P. gingivalis* cells standardized at OD_600_ of 2.0 with pre-warmed PBS at 37°C were mixed with laurdan reagent (Sigma-Aldrich, 40227) at a concentration of 10 µM by vortexing briefly, and afterward incubated for 10 min at 37°C. The cells were harvested by centrifugation, and washed four times with pre-warmed (37°C) laurdan buffer containing 137 mM NaCl, 2.7 mM KCl, 10 mM Na_2_HPO_4_, 1.8 mM KH_2_PO_4_, 0.2% glucose, and 1% N,N-dimethylformamide. Then, the laurdan-treated cells standardized at OD_600_ of 0.2 were mixed with a membrane fluidizer, benzyl alcohol, at a concentration of 150 mM by vortexing briefly. For endpoint spectroscopic analysis, 2 µL each of ME prepared at different concentrations or the vehicle control (DMSO) was mixed with 200 µL of the cell suspension in a well of a pre-warmed (37°C) black microtiter plate. The fluorescence measurement was performed (excitation: 350 nm, emission: 460 and 500 nm) by a fluorescence plate reader with a spectrum scanning mode (Cytation 5, BioTek).

### Membrane permeability and membrane potential assay

Bacterial membrane permeability and membrane potential in response to ME were examined by flow cytometry with TO-PRO-3 and DiOC_2_(3) used as a membrane-impermeable dye and a membrane potential indicator dye, respectively, as described previously (26). The membrane permeability of *P. gingivalis* cells was judged by the increase in the population of TO-PRO-3^+^ cells. Bacterial membrane potential was measured using *E. coli* BW25113 strain as a model bacterium, instead of *P. gingivalis*. Cells stained with DiOC_2_(3) can be visualized by flow cytometry with blue excitation (488 nm) and green (FL-1: 530 nm) or red (FL-3: 650 nm) emission filters. DiOC_2_(3) produces green fluorescence (FL-1: 530 nm) in all bacterial cells, then shifts to red (FL-3: 650 nm) in response to the increased membrane potential. The distribution of red/green fluorescence emitted by DiOC_2_(3)." to "The distribution of red/green fluorescence emitted by DiOC_2_(3) varies with change in ΔΨ. Data were analyzed by FACS Canto II (BD Biosciences Inc., Franklin Lakes, NJ, USA). All obtained data were analyzed with the FACS Diva software package (BD Biosciences Inc.).

### Autoaggregation assay

*P. gingivalis* cells cultured for 48 h were harvested by centrifugation. Two milliliters of whole cells standardized at OD_600_ = 1 after suspension in PBS was placed in a 14 mL polyethylene tube, and incubated at 4°C under static conditions. The OD_600_ of the phase above the sediment formed by aggregation was recorded at different time points.

### Clinical study

#### Study design

Patients with chronic periodontitis were assessed for eligibility at the Department of Periodontology (Nihon University Hospital School of Dentistry at Matsudo, Japan) between March 2021 and February 2022. Forty-five subjects who fulfilled the inclusion criteria were enrolled and randomly divided into the following three different groups: sodium azulene sulfonate hydrate, barley tea, or matcha. Inclusion criteria are as follows:

1. Patients with at least one tooth having PPD ≥5 mm after completion of initial periodontal therapy
2. No antimicrobial treatment in the last 3 months
3. No smoking experience
4. No allergy experience
5. Not younger than 20 years old

Patients received one of the three powders containing sodium azulene sulfonate hydrate, barley tea, or matcha. They dissolved the powder by themselves in tap water to prepare the mouthwash and gargled the mouthwash twice a day for a month, according to a trained periodontist’s instructions. Oral examination was performed and saliva samples were collected before and after the intervention. All 45 patients enrolled in the present study were followed up and analyzed. At the visit after intervention (Fig. 3B), we checked the records of individual administration, as well as interviewed the patients regarding whether mouth washing was properly performed. Adverse events were also documented with respect to onset, duration, treatment, relation to study medication, and outcome.

#### Matcha, azulene, and barley tea powders used for the intervention study

Matcha that was used for *in vitro* assays was used for the clinical study. Barley tea powder and azulene sulfonic acid powder were purchased from Sato-Foods Industries Co., Ltd. (Aichi, Japan), and TOYO Pharmaceutical Co., Ltd. (Tokyo, Japan), respectively. Matcha powder of 1.33 g (dry weight), barley tea powder of 1.49 g (dry weight), and azulene sulfonic acid of 2.0 g were sealed individually into laminated aluminum bags by Kyoeiseicha Co., Ltd. (Osaka, Japan). Patients were given matcha powder, barley tea powder, or azulene sulfonic acid, and the mouthwash was prepared at home by mixing with 20.7, 23.1, or 100 mL of tap water, respectively, to yield the mouthwashes at final concentrations of 64.3, 64.3, or 20 mg/mL, respectively. Each subject rinsed twice a day, morning and evening, with 10 mL of the respective mouthwash. After rinsing, eating and drinking were prohibited for 30 min.

#### Clinical parameters

Clinical parameters of subjects were recorded by periodontal specialists who were certified by the Japanese Society of Periodontology and trained in Department of Periodontology at Nihon University Hospital School of Dentistry at Matsudo. All the examiners were blinded to the type of treatment received by the subject. The following clinical parameters were measured for each subject: plaque index (Pl.I.), gingival index (GI), PPD, BOP, periodontal epithelial surface area (PESA), and periodontal inflamed surface area (PISA). The PPD was measured from the gingival margin to the tip of inserted probe using a periodontal probe with 1 mm markings. Measurements were taken at six sites per tooth and the nearest whole millimeter measurement was recorded if readings fell between markings. Following the PPD measurement, bleeding was judged by the presence or absence of BOP at six sites per tooth. The BOP was recorded as “0” or “1,” when no bleeding occurred or bleeding occurred within 10 s after probing, respectively. PESA and PISA were calculated according to the method of Nesse et al. (29).

#### Sample collection, DNA extraction, and real-time PCR

At the first visit, trained periodontists instructed the subject on how to collect saliva. Before and after the intervention, each subject collected more than 5 mL of whole saliva stimulated by chewing gum (SalivaGum-α, Tokyo Shizai Co., Tokyo, Japan) at the hospital. All saliva samples were stored and transported at 4°C to a laboratory of the National Institute of Infectious Diseases, where the total bacterial DNAs were extracted from the saliva samples within 48 h after saliva collection, as described previously (30). Real-time PCR analysis was performed to detect the presence of the following six pathobionts using the salivary DNA as a template: *P. gingivalis, Tannerella forsythia, Treponema denticola, Fusobacterium nucleatum, Aggregatibacter actinomycetemcomitans,* and *Prevotella intermedia*. For the real-time PCR reaction, we used TB Green Premix Ex Taq II (Tli RNaseH Plus) (Takara Bio, Shiga, Japan), universal or six species-specific primers (Table S1), and the StepOne Real-Time PCR System (Thermo Fisher Scientific). The reaction mixture recipe used in this study is as follows: 1 µL of DNA was added to 9 µL of premix solution containing 1× TB Green Premix Ex Taq II, 0.2 µM of each primer, and 1× ROX Reference Dye II. The PCR thermal conditions are as follows: 1 cycle of 3 min at 95°C followed by 40 cycles of 5 s at 95°C and 34 s at 60°C.

#### Primary and secondary endpoints

The primary endpoint of this study was improvement of the following oral examination parameters by the intervention: Pl.I., GI, PPD, BOP, PISA, and PESA. The secondary endpoints were clearance of oral pathobionts in the oral cavity after the 1-month mouthwash intervention.

#### Stopping criteria

The parameters for stopping the intervention trial are as follows:

1. When the patient declined to participate in the study or withdrew the consent
2. When the patient’s compliance became significantly impaired due to worsening of the patient’s medical conditions.
3. When the examiner deemed it appropriate to discontinue the study for other reasons

### Statistical analysis

Statistical analysis was performed using a Mann-Whitney U-test or one-way analysis of variance followed by Dunnett’s multiple comparison test. *P*-values of 0.05 or less were considered to be statistically significant.

## RESULTS

### Major periodontal pathobiont *P. gingivalis* shows highest sensitivity among oral bacteria

**Fig 1:**
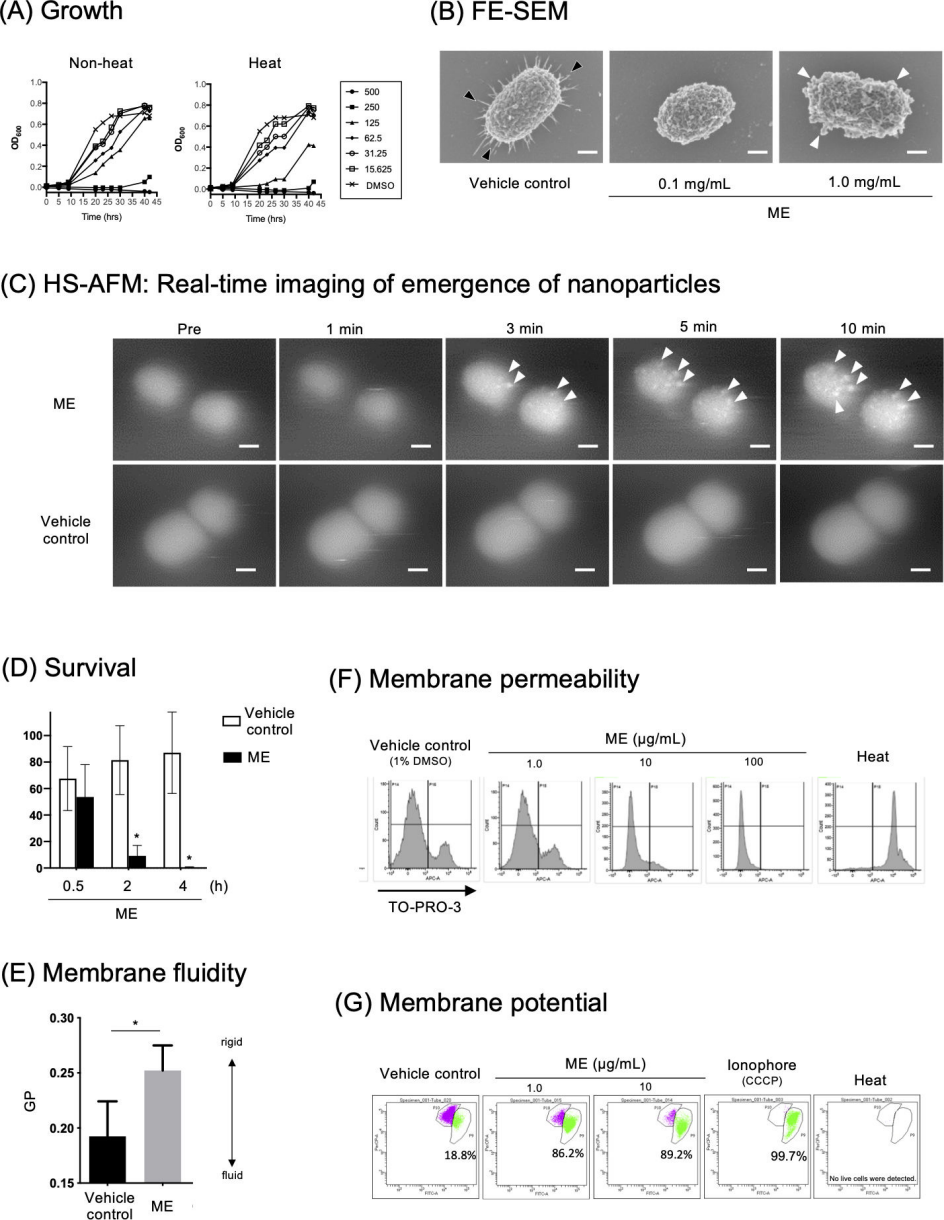
ME inhibits *P. gingivalis* growth by triggering both morphological and physiological changes at the cell envelope. (**A**) Growth inhibition. *P. gingivalis* was anaerobically grown for 2 days in the absence or presence of ME (left) or heated ME (right) at different concentrations (from 15.625 to 500 µg/mL). In the vehicle control, 1% DMSO was added instead of the samples. The turbidity (OD_600_) of the culture broth was measured at different time points. (**B**) FE-SEM. *P. gingivalis* was treated with ME at concentrations of 100 µg/mL or 1 mg/mL and vehicle control (1% DMSO) for 30 min at 18°C to 25°C. Morphology of *P. gingivalis* cells was observed by FE-SEM. Fimbriae and nano-particle formation are denoted by black and white arrowheads, respectively. Bars: 150 nm. (**C**) HS-AFM. Spatiotemporal analysis of *P. gingivalis* cell envelope after treatment with ME (1 µg/µL) or vehicle control (1% DMSO). Nano-particles are denoted by white arrowheads. Bars: 200 nm. See also Video S1. (**D**) Time-course killing activity. *P. gingivalis* cells were treated with vehicle control (Control) and ME at 100 µg/mL for 0.5, 2, and 4 h. Survival rates were evaluated by counting CFUs on BAPs and was denoted as [CFU (tested sample)/CFU (baseline, untreated sample)] × 100 (%). *P < 0.05. (**E**) Membrane fluidity. Fluidity of *P. gingivalis* cell membrane was estimated by a fluorescence probe, laurdan, that intercalates into the membrane bilayer and displays an emission wavelength shift depending on the amount of water molecules in the membrane. *P. gingivalis* cells were treated with ME at a concentration of 100 µg/mL. The membrane fluidity of *P. gingivalis* was calculated as generalized polarization of laurdan (GP) according to the formula: GP = (A_455_ − A_495_)/(A_455_ + A_495_). (**F**) Membrane permeability. The membrane permeability of *P. gingivalis* was estimated by the membrane impermeable dye TO-PRO-3 using flow cytometry. The TO-PRO-3-positive *P. gingivalis* cell population inversely correlated to the concentrations of ME. *P. gingivalis* cells were also subjected to heat treatment (Heat) as a control of cells with increased membrane permeability. (**G**) Membrane potential. A membrane potential-sensitive dye DiOC_2_(3) was used for Gram-negative bacterial membrane potential assay using *E. coli* as a model. The intensity of the ionophoric activity was estimated by the increased green-colored and decreased purple-colored populations, indicating depolarized and polarized cell populations, respectively, as shown in each dot plot panel. The percentages shown are calculated as a ratio of the number of depolarized cells to the number of total cells that retained membrane integrity. No data of membrane potential were obtained in heated cells, as heat treatment resulted in loss of the membrane integrity of all the cells. ME was used at a concentration of 1 or 10 µg/mL. carbonyl cyanide 3-chlorophenylhydrazone (CCCP), a strong ionophore, was also used as a positive control triggering membrane depolarization. Cells were also subjected to heat treatment (Heat) as a control of cells with increased membrane permeability.

ME obtained using H_2_O-acetone solvent was used in a growth inhibition assay against 16 oral bacterial species, including three strains of *P. gingivalis*. The MICs of ME are shown in Table 1 with Socransky’s community ordination (4), in which bacterial species belonging to the “red” or “orange” complex are primarily or secondarily related to periodontitis, respectively, while the “yellow” complexes are associated with periodontal health. *P. gingivalis* (red complex), *Prevotella nigrescens* (orange), and *Fusobacterium nucleatum* (orange) had higher sensitivities to ME (125–250 µg/mL) than other oral bacterial species (>500 µg/mL), including representative oral commensals, streptococci (yellow), and *Aggregatibacter actinomycetemcomitans* serotype b (not allocated: NA). ME inhibited growth of *P. gingivalis* strain ATCC 33277 in a dose-dependent manner (Fig. 1A). The growth inhibition activity was still retained by ME treated at 100°C for 20 min (Fig. 1A), suggesting this activity was attributed to heat-stable component(s). In addition, nearly all *P. gingivalis* cells were killed by ME within 2 h (Fig. 1D; Fig. S1A), and all cells were killed by 4 h (Fig. 1D), suggesting that ME interfered with the growth and exerts a bactericidal activity.

The high selectivity of ME toward *P. gingivalis* raised the possibility that ME might inhibit the growth through a gingipain- or a T9SS-mediated mechanism. The T9SS of *P. gingivalis* is responsible for producing “a virulence coat” on the cell surface and outer membrane vesicle surface, which are composed of a wide range of virulence factors, including anionic lipopolysaccharide, gingipains, hemagglutinin HBP35, zinc carboxypeptidase CPG70, peptidylarginine deiminase, among others (31, 32). So, we also examined MICs of ME against a three gingipains-deficient mutant KDP981 (*rgpA*^−^ *rgpB*^−^ *kgp*^−^) and a T9SS-deficient KDP355 (*porK*^−^). The results showed that the MICs of the *rgpA^−^ rgpB^−^ kgp*^−^ and *porK*^−^ strains were 125 and 62.5 µg/mL, respectively (data not shown). The findings suggested that interaction between *P. gingivalis* and ME is mediated neither by gingipains nor by the other T9SS substrates on the bacterial surface, but rather, the lack of the virulence coat might enhance the accessibility of ME to the cell envelope structure of *P. gingivalis*.

### ME triggers morphological and physiological changes at the *P. gingivalis* envelope

In FE-SEM analysis (Fig. 1B), typical fimbrial structures radiating out from *P. gingivalis* cells (denoted by black arrowheads, vehicle control) disappeared after treatment with ME at the concentrations of 0.1 mg/mL. In addition, the emergence of nano-particle structures was clearly observed when cells were treated with 1.0 mg/mL ME (Fig. 1B, denoted by white arrowheads). HS-AFM imaging revealed that these nano-particle structures ranging in size from 20 to 30 nm in diameter appeared on the cell surfaces at 3 min after ME addition, and then gradually increased in size up to ca. 50 nm for 10 min (Fig. 1C; Video S1). These results are in good agreement with those obtained by our previous studies, in which aberrant membrane blebs were developed on the cell surface of *P. gingivalis* following treatment with extracts of propolis (25), curry leaf (33), and fennel (26). In general, outer membrane blebbing are induced due to the structural change of the cell envelope, e.g., by a decrease in outer membrane-peptidoglycan cross-linking proteins (34), an intercalation compound that induced membrane curvature as molecular wedges (35), or the accumulation of misfolded proteins in the periplasm (36). We speculated that ME might induce outer membrane fluctuation with change in physicochemical dynamics of the bacterial envelope. To elucidate the mechanism of the ME-mediated nano-particle formation, bacterial membrane physiology in response to ME addition was also assessed. The membrane fluidity of *P. gingivalis* was examined using the fluorescent dye laurdan (Fig. 1E), which intercalates into the membrane bilayer depending on the amount of water molecules in the membrane (27, 28). The findings showed that membranes treated with ME become stiffer compared to those treated with vehicle control, and the rigidity of the membrane was enhanced by ME in a dose-dependent manner (Fig. 1E; Fig. S1B). In addition, an indicator dye of membrane integrity, TO-PRO-3, did not permeate to *P. gingivalis* cells following ME treatment (Fig. 1F), and ME rather interfered with TO-PRO-3 internalization, demonstrating that *P. gingivalis* membrane permeability was decreased by addition of ME. We also found that ME triggered membrane depolarization in a DiOC_2_(3)-*E. coli* model (Fig. 1G). These findings demonstrate that ME dramatically induced both morphological and physiological changes at the envelope of *P. gingivalis* cells in the process of a bactericidal event.

### ME induces autoaggregation of *P. gingivalis*

**Fig 2:**
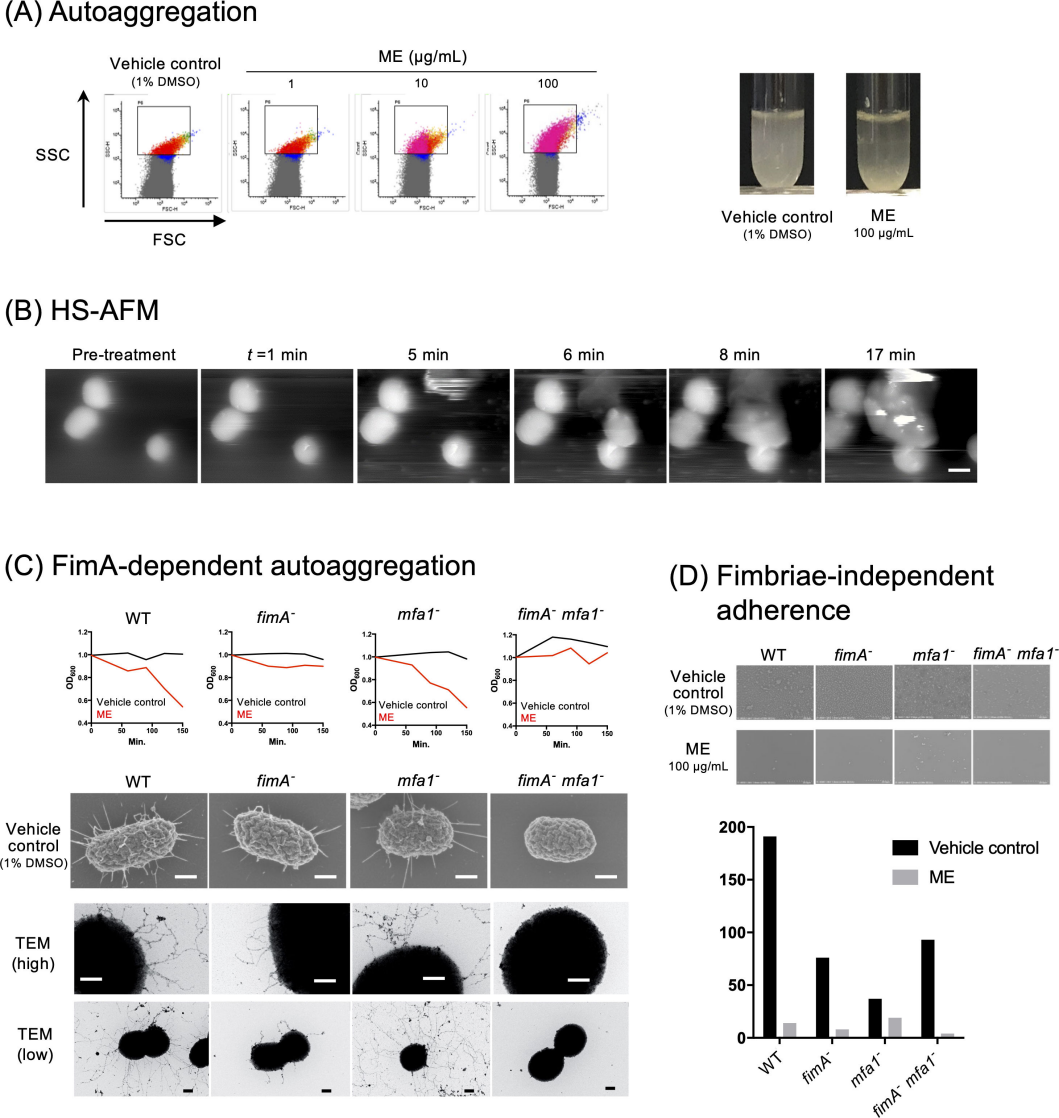
Aggregation and adherence of *P. gingivalis* treated with ME. (**A**) *In vitro* autoaggregation assay. In flow cytometry analysis (left), *P. gingivalis* cells were treated with vehicle control (1% DMSO) or ME at different concentrations (1, 10, and 100 µg/mL). The *x*- and *y*-axes showed the FSC and the SSC, respectively. Shown (right) are the difference in turbidity of the tubes 2 h after treatment with vehicle control (1% DMSO) and ME (100 µg/mL) in *in vitro* tube assay. (**B**) Real-time HS-AFM imaging of aggregation of *P. gingivalis* cells after ME treatment (1.0 mg/mL). Bar: 300 nm. See also Video S2. (**C**) FimA-dependent autoaggregation. *In vitro* aggregation assays were performed using *P. gingivalis* WT, FimA mutant (*fimA*^−^), Mfa1 mutant (*mfa1^−^*), and FimA and Mfa1 double mutant (*fimA*^−^ *mfa1^−^*) strains in the presence or absence of ME. The top panels showed the change in turbidity (OD_600_) for 150 min. The morphology of each *P. gingivalis* cell (FE-SEM) treated without or with ME at a concentration of 1 mg/mL is also shown in the middle or bottom panel, respectively. Bars: 200 nm. (**D**) Fimbriae-independent adherence. ME dramatically decreased the adherence of cells not only of wild type but also of a series of fimbrial mutant strains.

Using flow cytometry of *P. gingivalis* cells, we observed that treatment with ME increased the values of both the forward scatter (FSC) and the side scatter (SSC) in a dose-dependent manner (Fig. 2A, left panels), suggesting a possible ME-mediated autoaggregation. ME-mediated autoaggregation was confirmed by *in vitro* tube assay (Fig. 2A, right panel), and HS-AFM analysis revealed aggregation of *P. gingivalis* when the cells were treated with ME (Fig. 2B; Video S2). Real-time imaging showed some *P. gingivalis* agglutination with cells migrated from another area; the autoaggregation occurred from 5 min to 17 min in the movie (Fig. 2B; Video S2). Furthermore, using an *in vitro* autoaggregation assay with the 33277 (wild type, WT) strain and the isogenic fimbriae mutants, we found that ME-mediated autoaggregation was induced by WT and *mfa1*^−^ strains that possessed the major fimbriae FimA, but was not induced by two FimA fimbriae-deficient strains (*fimA*^−^ and *fimA*^−^ *mfa1^−^* strains) (Fig. 2C). In contrast, the minor fimbriae Mfa1 was not involved in the ME-mediated aggregation (Fig. 2C). Together, the data suggested that FimA is responsible for ME-mediated *P. gingivalis* aggregation. We also examined the effect of ME on adherence of *P. gingivalis* on the poly-L-lysine-coated slide glass. FE-SEM results clearly showed that ME inhibited the colonization on the slide glass surface (Fig. 2D). The inhibition of colonization was happening irrespective of the presence of FimA or Mfa1 (Fig. 2D).

### Significance of pyrogallol-type B-ring of catechin for growth inhibition of *P. gingivalis*

The multi-step fractionation/purification of ME (Fig. S2A and B) isolated nine matcha-derived catechins (Table 2). We investigated both their autoaggregation and growth inhibition activities. All catechins showed autoaggregation activity against *P. gingivalis* (Table 2), suggesting that catechins non-specifically enhance autoaggregation irrespective of differences in their functional groups. In a growth assay, the pyrogallol-type B-ring in catechins, e.g., EGCG (M-7) and epigallocatechin (EGC, M-4), strongly inhibited *P. gingivalis* growth, while no growth inhibitory activity was observed with catechin (M-1), epicatechin (M-2), or epicatechin-3-*O*-gallate (M-5) treatment. The data showed a structure-activity correlation between pyrogallol-type B-ring in catechins and its growth inhibitory activity. On the other hand, compositional analysis of matcha revealed that it is enriched with catechins possessing the pyrogallol-type B-ring, which accounted for 61.7%–76.9% of total catechins (Fig. S4). The ratio of the sum of EGCG (M-7) and EGC (M-4) to total matcha catechins was much higher than that of ordinary green tea (Fig. S4). These findings motivate further clinical studies to assess the therapeutic applicability matcha for possible elimination of *P. gingivalis* in the oral cavity.

**TABLE 2:** Matcha catechin components with MIC and aggregation activity

ID	Catechin	Description (molecular weight/functional group)	Content (%)^*a*^	MIC (µM)^*b*^	Aggregation activity^*c*^
M-1	Catechin	290.26	0.3	>500	69.7%
M-2	Epicatechin	290.26/Epi (+)	3.5	>500	70.2%
M-3	Gallocatechin	306.27/Gallo (+)	1.2	500	68.1%
M-4	Epigallocatechin	306.27/Epi (+), Gallo (+)	18.5	250	68.3%
M-5	Epicatechin-3-*O*-gallate	456.40/Epi (+), 3-*O*-gallate (+)	8.6	>500	89.0%
M-6	Gallocatechin-3-*O*-gallate	456.40/Gallo (+), 3-*O*-gallate (+)	0.7	250	NT
M-7	Epigallocatechin-3-*O*-gallate	458.37/Epi (+), Gallo (+), 3-*O*-gallate (+)	57.3	500	72.1%
M-8	Epigallocatechin-3-*O*-(3"*O*-methyl)-gallate	472.40/Epi (+), Gallo (+), 3-*O*-(3"-*O*-methyl)-gallate (+)	ND	500	NT
M-9	1,4,6-tri-*O*-galloyl-glucose	636.47/Gallo (+++)	ND	500	NT
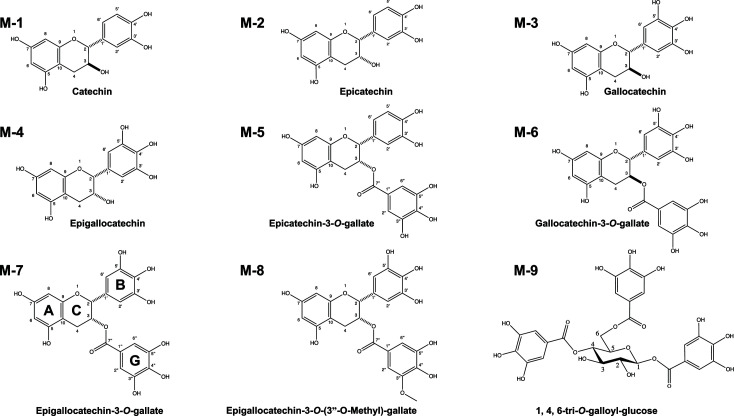					

^*a*^ Contents of catechins (%) in matcha were analyzed using micellar electrokinetic chromatography as described in reference 37.

^*b*^ MIC (µM) was determined by microdilution.

^*c*^ Aggregation activity (%) was calculated as 100 × [the value of A_600_ 2 h after addition of each catechin/the value of OD_600_ 2 h after addition of the vehicle control (DMSO)].

### Matcha mouthwash reduces the number of *P. gingivalis* in saliva

**Fig 3:**
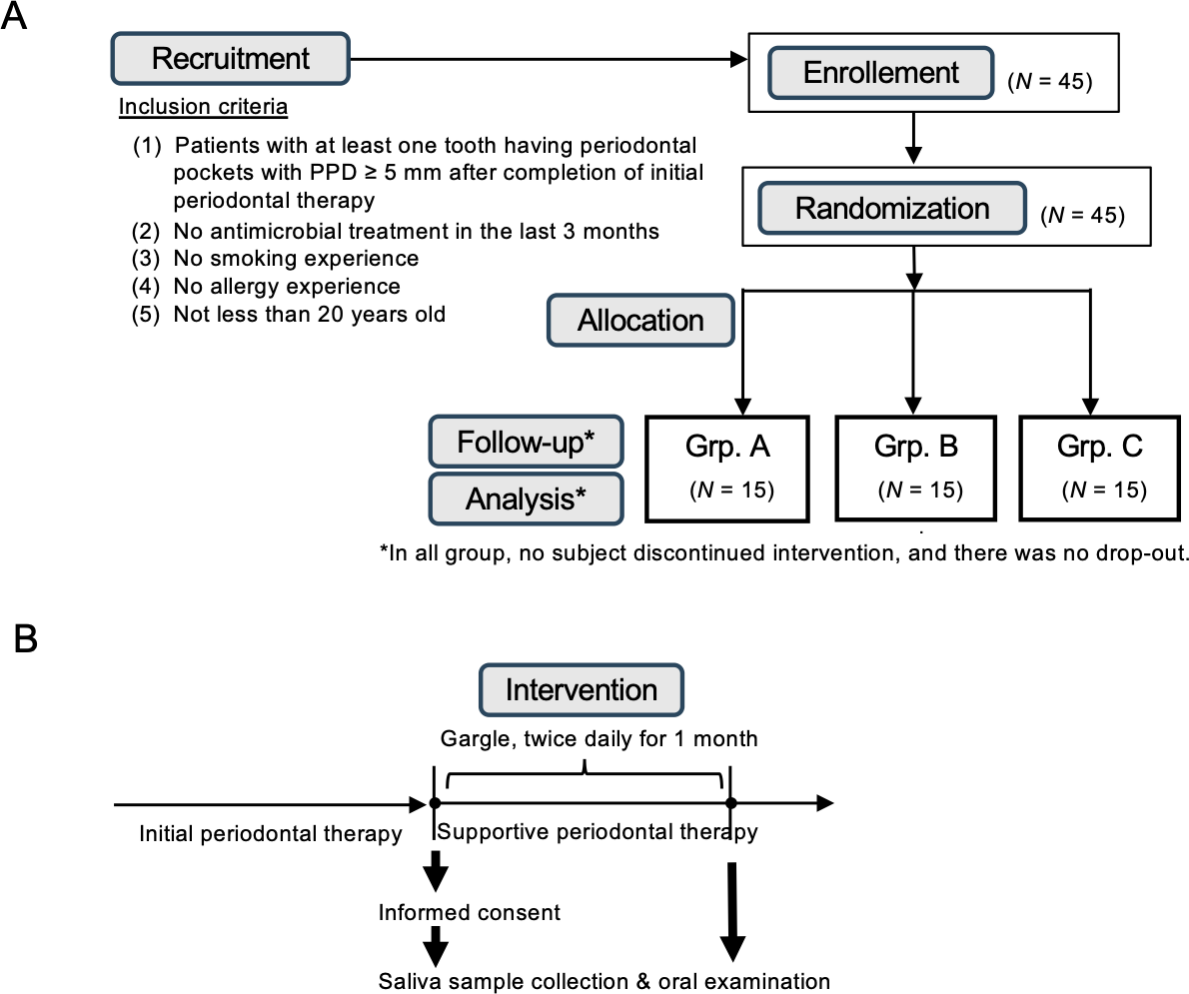
A clinical study to examine the effect of matcha mouthwash on periodontal diseases. (**A**) Flow diagram of study protocol. Forty-five subjects who fulfilled the inclusion criteria were enrolled between March 2021 and February 2022. They were randomly divided into three groups, and given a powder containing one of the following three different agents: sodium azulene sulfonate hydrate (Grp. A), barley tea (Grp. B), or matcha (Grp. C). All 45 patients enrolled in the present study were followed up and analyzed, i.e., there were no dropouts. (**B**) Visit protocol. Informed consent was obtained from all the patients who completed initial periodontal therapy. The intervention was performed during the period of supportive periodontal therapy for each patient. The patients prepared the mouthwash with tap water by themselves, and used it twice a day for 1 month, according to the dentist’s instructions. Oral examination was performed and saliva samples were collected before and after the intervention.

**Fig 4:**
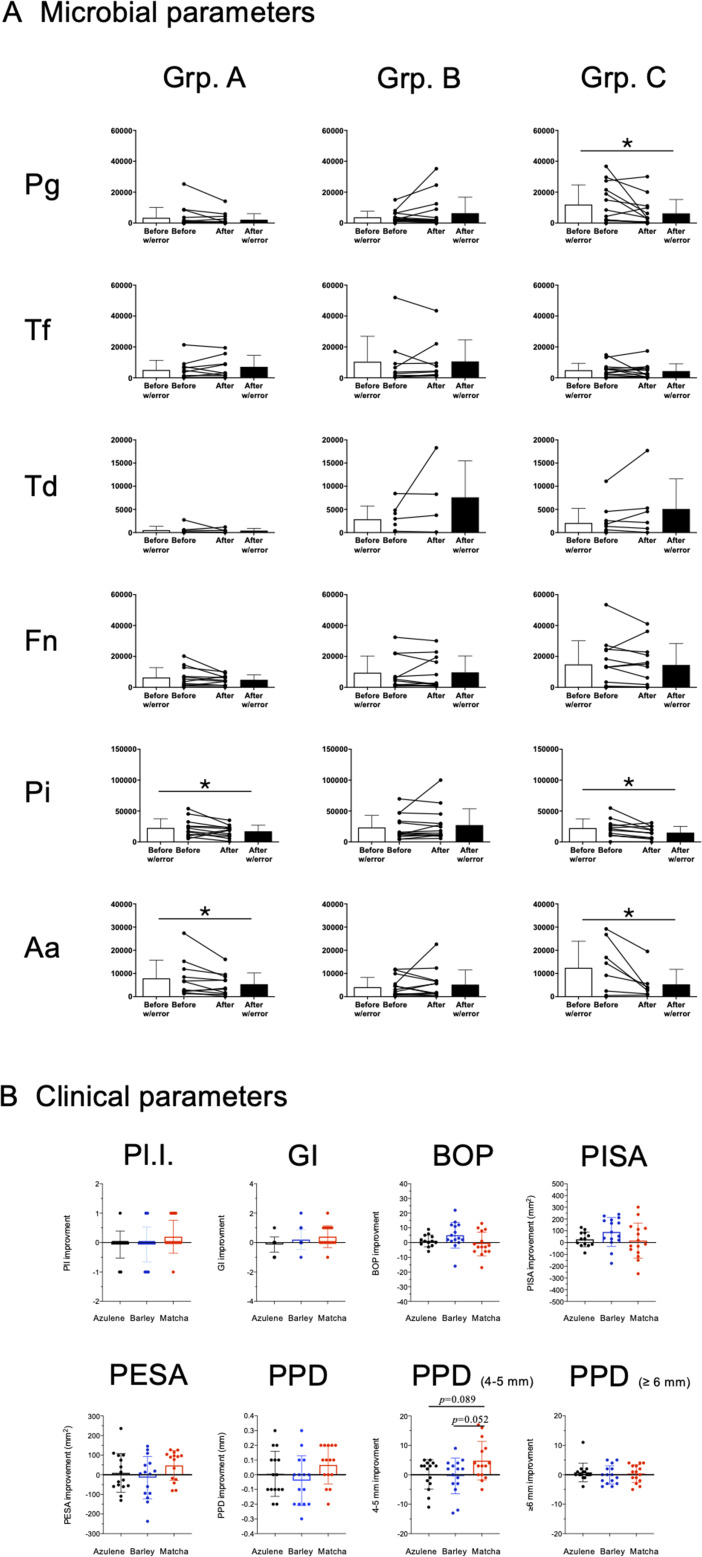
(A) Effect of gargling with different ingredients on periodontal pathobionts in saliva. The number of the following six bacterial species in saliva was quantified by real-time PCR: *P*. *gingivalis* (Pg), *Tannerella forsythia* (Tf), *Treponema denticola* (Td), *Fusobacterium nucleatum* (Fn), *Prevotella intermedia* (Pi), and *Aggregatibacter actinomycetemcomitans* (Aa). Asterisks denote P < 0.05. (B) The effect on eight different parameters associated with periodontal tissues. Improvement of the following clinical parameters was compared between the groups: Pl.I., GI, BOP, PISA, PESA, PPD, PPD between 4 and 5 mm (PPD [4–5 mm]), PPD 6 mm or greater (PPD [≥ 6 mm]).

In a pilot intervention study, the microbiological and therapeutic effects of matcha mouthwash were assessed in patients with periodontitis. A flow diagram of the study protocol and timeline of the clinical trial are shown in Fig. 3A and B, respectively. No adverse events or complications were recorded during the study. The yield and purity of DNA isolated from saliva samples was confirmed by real-time PCR to detect the universal region of the bacterial 16s rRNA gene, in which the copy numbers were relatively comparable between the samples (Fig. S5). The effect of matcha mouthwash on periodontal pathobionts is shown in Fig. 4A. Although some patients who used matcha mouthwash did not change the numbers of *P. gingivalis*a by unknown mechanism, the number of *P. gingivalis* in saliva was significantly reduced by matcha mouthwash compared to the pre-intervention level (Fig. 4B, *P* < 0.05). The numbers of *P. intermedia* and *A. actinomycetemcomitans* were significantly reduced in both the matcha and the azulene mouthwash groups, when compared to their respective pre-intervention levels (Fig. 4B, *P* < 0.05). A tendency toward improvement in teeth with 4 to 5 mm PPD (4–5 mm) was observed in the matcha group when compared to the azulene group (*P* = 0.089) or the barley tea group (*P* = 0.052), albeit the differences were not statistically significant (Fig. 4B).

## DISCUSSION

In *in vitro* growth inhibition assay, ME was found to show a narrow-spectrum antimicrobial effect on important periodontal pathobionts such as *P. gingivalis*, but not on oral streptococcal strains (Table 1), which are associated with periodontitis (4) and oral dysbiosis (8). We hypothesized that topical application of matcha may eliminate periodontal pathobionts via maintaining benign microorganisms such as oral streptococci, without inducing oral dysbiosis. To test this hypothesis, we performed a clinical intervention trial with matcha mouthwash on patients with periodontitis. The numbers of *P. gingivalis* in saliva were significantly reduced by matcha mouthwash compared to the pre-intervention level, while there was no statistically significant difference in the numbers of *P. gingivalis* in the other two control groups, i.e., those who used sodium azulene sulfonate or barley tea mouthwash. Among all the clinical parameters examined in this study, a tendency toward improvement in teeth with 4 to 5 mm PPD (4–5 mm) was observed in the matcha group compared to the azulene group (*P* = 0.089) and the barley tea group (*P* = 0.052). However, no improvement was observed for PPD 6 mm or greater (PPD [≥6 mm]) in the matcha group. Therefore, it may be that the mouthwash was only effective for shallow periodontal pockets (≤5 mm), not for deep periodontal pockets (≥6 mm). More effective drug delivery into the bottom of periodontal pockets through drug formulations (e.g., gel or ointment to retain drug longer) and administration methods (e.g., injection using a syringe with a soft needle-type tip) may improve periodontitis by allowing the drug to reach deeper into periodontal pockets where the bacteria reside.

ME-induced *P. gingivalis* growth inhibition (Fig. 1A) was accompanied by both morphological (Fig. 1B and C; Fig. S1 ) and physiological changes at bacterial envelopes with decreased membrane fluidity (Fig. 1E) and membrane depolarization (Fig. 1G) but without loss of membrane integrity (Fig. 1F). These findings are highly consistent with a recent report on bacterial membrane physiology (38), in which even very low membrane fluidity allowed robust diffusion barrier function, but interfered with essential cellular processes, including cytokinesis, envelope expansion, chromosome replication/segregation, and maintenance of membrane potential. In another report, experimental measurements and mathematical modeling indicated that rates of respiratory metabolism and cell growth were dependent on membrane viscosity and its effects on diffusion (39). Therefore, the ME-driven decrease in bacterial membrane fluidity may have resulted in *P. gingivalis* growth inhibition by decreasing respiratory function via blocking the electron transport chain.

Capsular polysaccharides in some bacteria are often essential for antimicrobial resistance. However, this was not the case for *P. gingivalis*. As shown in Table 1, not only a non-capsulated *P. gingivalis* strain (ATCC 33277, MIC: 250 µg/mL) but also two encapsulated strains (W50, 250 µg/mL and W83, 125 µg/mL) were susceptible to ME at relatively low concentrations. We therefore ruled out the involvement of capsular polysaccharides of *P. gingivalis* in ME susceptibility. On the other hand, oral streptococcal strains and *A. actinomycetemcomitans* were resistant to ME, while *Porphyromonas* and *Prevotella* were sensitive to ME (Table 1). Both *Porphyromonas* and *Prevotella* possess the T9SS, which is responsible for producing “a virulence coat” on the cell surface and outer membrane vesicles. The virulence coat of *P. gingivalis* is known to contain a wide range of virulence factors, including gingipains, anionic lipopolysaccharide, and so on (31, 32). To elucidate whether gingipains and the other T9SS substrates are associated with the ME-mediated growth inhibition, we examined whether the MICs of ME change in the gingipain and T9SS mutant strains. The results showed that both the gingipain-deficient (MIC, 125 µg/mL) and T9SS-deficient (62.5 µg/mL) strains showed higher susceptibilities to ME as compared to the parental strain ATCC 33277 (250 µg/mL). In our previous study, the T9SS-deficient strain (*porK*^-^) that lack the virulence coat dramatically altered the cell surface morphology of *P. gingivalis* (40). Taken together, the virulence coat including gingipains is not the primary target of ME against *P. gingivalis*, but rather may protect from ME-mediated inhibitory action by blocking the accessibility of ME to *P. gingivalis*. On the other hand, gingipain activity has been reported to be abolished by green tea- and apple-derived polyphenol (41, 42). So, the high susceptibility of *P. gingivalis* to ME may be in part related to the ability of component(s) of matcha to inhibit gingipain activities. We therefore speculate that the mechanism of ME-mediated inhibition against *P. gingivalis* is based on both the membrane-targeting activity and the activity to eliminate the gingipain activity.

Component analysis of matcha polyphenols confirmed that EGCG (M-7) and EGC (M-4) were two predominant catechins in matcha, and the ratio of the sum of the two compounds was much higher than that for ordinary green tea (Fig. S4). Both EGCG and EGC, which are enriched in matcha, showed strong antimicrobial activity against *P. gingivalis* (Table 2). From these findings, we propose that matcha has an advantage over ordinary green tea due to its therapeutic activity against *P. gingivalis* infection. Several previous reports showed that EGCG inhibited the growth of *P. gingivalis* (11, 15), which is in good agreement with our findings (Table 2). On the other hand, there was a discrepancy in the interpretation of the structure-activity relationship of catechins between one of the previous studies and our study. In the MIC determination of green tea catechins against three *P. gingivalis* strains, Sakanaka et al. showed that EGCG was more effective than other catechins including catechin, epicatechin, gallocatechin, epigallocatechin, and epicatechin gallate (11). However, our study showed that growth inhibition of *P. gingivalis* was due to the galloyl group of B-ring of catechin. We have also shown that the presence of the *O*-gallate structure had no effect on growth inhibition against *P. gingivalis*. On the other hand, the presence of the *O*-gallate structure was indispensable for growth inhibition of another periodontal pathobiont, *Eikenella corrodens* (43), suggesting that the relationship between the growth inhibitory effect and the chemical structure of catechins should be determined on a pathogen-specific basis. Given that the ME contains a wide range of polyphenols that exert different effects on *P. gingivalis*, it is highly likely that the multimodality is driven by additive or synergistic antimicrobial effect of different ME-derived compounds.

The current study is not the first to show that tea-derived catechins have an antimicrobial effect on *P. gingivalis*. However, our results suggest prophylactic and therapeutic potential for matcha as a multimodal drug for periodontal diseases. Furthermore, our comprehensive analysis of those antibacterial activities toward *P. gingivalis* not only revealed how they affect the envelope of *P. gingivalis* but also provided mechanistic insight into the ability to trigger autoaggregation of *P. gingivalis* cells. In addition, the present intervention clinical trial resulted in beneficial outcomes. Nevertheless, our clinical study has several limitations. For example, no consideration was given to the patient’s genetic predisposition to periodontal disease. It should be also noted that several confounding factors could have affected these results, e.g., how many times and how long the individuals brushed their teeth and used dental floss, what kind of toothpaste to use, and so on. Another limitation was based on the administration method. In the present study, the clinical outcomes of matcha were tested only by means of mouthwash. It remains unknown to what extent the mechanical action of mouthwash itself reduces the number of bacteria in oral cavity. It was also unfortunate that our ethical committee did not allow to test the clinical effect by an alternative delivery option, e.g., by injection to periodontal pockets using a syringe with a soft needle-type tip, by which matcha could directly reach to the bottom of periodontal pockets. Finally, the present clinical trial lacked a mock-mouthwash control group, because of ethical inability to create a group that receives a mouthwash (water) without any ingredient. Therefore, it is essential to perform another clinical trial aiming to find suitable delivery method into periodontal pockets in the future. Furthermore, a larger-scale clinical trial of topical matcha is needed to confirm its clinical efficacy and benefit to periodontitis patients. Despite the limitations to be considered, our findings may pave the way for a promising, novel therapeutic option of using matcha for treatment to treat patients with periodontitis.

## SUPPLEMENTAL MATERIAL

Supplemental material spectrum.03426-23-s0001.docx Fig. S1 to S6; Table S1.

Video S1 spectrum.03426-23-s0002.avi Real-time observation of cells after treatment with ME at 1 mg/mL.

Video S2 spectrum.03426-23-s0003.avi Real-time observation of cells after treatment with ME at 0.1 mg/mL.

ASM does not own the copyrights to Supplemental Material that may be linked to, or accessed through, an article. The authors have granted ASM a non-exclusive, world-wide license to publish the Supplemental Material files. Please contact the corresponding author directly for reuse.
