# Supplementary material for: Multimodal inhibitory effect of matcha on Porphyromonas gingivalis
Source: Microbiol Spectr. 2024 May 21;12(7):e03426-23. doi: 10.1128/spectrum.03426-23 (PMC11218439; doi:10.1128/spectrum.03426-23)
Supplement: Supplemental material — Fig. S1 to S6; Table S1. [file spectrum.03426-23-s0001.docx]

**Supplemental File**

**Multimodal inhibitory effect of matcha on *Porphyromonas gingivalis***

**Ryoma Nakao^a#^, Ayami Takatsuka^a,b^, Kengo Mandokoro^b^, Naoki Narisawa^b^, Tsuyoshi Ikeda^c^, Hideki Takai^d^, Yorimasa Ogata^d^**

^a^ Department of Bacteriology I, National Institute of Infectious Diseases, Tokyo, Japan. ^b^ Department of Food Bioscience and Biotechnology, College of Bioresource Science, Nihon University, Kanagawa, Japan.

^c^ Department of Pharmaceutical Sciences, Sojo University, Kumamoto, Japan.

^d^ Department of Periodontology, Nihon University School of Dentistry at Matsudo, Chiba, Japan.

^#^Address correspondence to: Ryoma Nakao, ryoma73@niid.go.jp.

**Contents**

**Supplemental Figure 1**

**Supplemental Figure 2**

**Supplemental Figure 3**

**Supplemental Figure 4**

**Supplemental Figure 5**

**Supplemental Figure 6**

**Supplemental Table 1**

**Supplemental Movie 1**

**Supplemental Movie 2**

**Supplemental Figure 1: Effect of ME on Pg cell viability and membrane fluidity.**

(A) Dose-dependent killing activity. Pg cells were treated without (Vehicle control) and with ME at different concentrations for 2 hours. Survival rates were evaluated by counting CFUs on BAPs and were denoted as [CFU (tested sample) / CFU (Baseline, untreated sample)] x 100. (E) Membrane fluidity. Fluidity of Pg cell membrane was estimated by a fluorescence probe, laurdan, that intercalates into the membrane bilayer and displays an emission wavelength shift depending on the amount of water molecules in the membrane. In the left panel, a typical fluorescence spectrum analysis was shown with appearance of the two peaks at 455 and 495 nm, denoted by black arrowheads. The Y axis shows relative fluorescence intensity unit (RFU) of laurdan at each wave-length. The membrane fluidity of Pg was calculated as generalized polarization of laurdan (GP) according to the formula: GP = (A_455_ - A_495_)/(A_455_ + A_495_). In the right panel, Pg cells were treated with ME at different concentrations ranging from 0.1 to 100 µg/mL.

**Supplemental Figure 2: Compound isolation from Matcha**

(A) Flow-1: Compound isolation from matcha. Matcha powder was ultra-sonicated for four hours in H_2_O-aceton solution (1:1), to yield matcha extract (ME). Then, the methanol (MeOH) fraction was collected from ME by using an MCI gel CHP-20P. The MeOH was fractionated into Fr. 1 to 9. Fr. 2, which was the enriched catechin group, was also further fractionated into nine fractions (Fr. 2-1 to 2-9). Finally, catechin, epicatechin, gallocatechin, epigallocatechin, and epigallocatechin-3-*O*-gallate were isolated from Fr. 2-4-1, 2-4-2, 2-5-1, 2-6-1, and 2-8-1, respectively. Fr. 4 was further fractionated according to the process overview described in Fig. 2 (B). (B): Flow-2: Fractionation and compound isolation from Fr. 4. Fr. 4 was fractionated into 11 fractions (Fr. 4-1 to 4-11) using Sephadex LH-20. Fr. 4-10 and Fr. 4-11 was further fractionated into three fractions (Fr. 4-10-1 to 4-10-3) and three fractions (Fr. 4-11-1 to 4-11-3), respectively using HPLC.　Finally, tri-*O*-galloyl-glucose, epigallocatechin-3-*O*-(3”-*O*-Methyl)-gallate, epicatechin-3-*O*-gallate, epigallocatechin-3-*O*-gallate, gallocatechin-3-*O*-gallate, and epicatechin-3-*O*-gallate were isolated from Fr. 4-10-1, 4-10-2, 4-10-3, 4-11-1, 4-11-2, and 4-11-3, respectively.

**Supplemental Figure 3: Growth assay of Pg in the presence of nine compounds (M-1 to M-9).**

Growth of *P. gingivalis* in the presence of nine compounds (M-1 to M-9) was monitored as the turbidity (OD_620_) of the bacterial culture for 48 hours.

**Supplemental Figure 4:** **Contents of major nutrients and polyphenols in green tea and matcha.**

The composition of Matcha (n = 3) were compared with that of of green tea extract (n = 2). Matcha lots-1, 2, and 3 were harvested in 2019, 2020, and 2022, respectively, Green tea extract lots-1 and 2 were harvested in 2019 and 2018, respectively, Contents of major macro nutrients (left) and polyphenols (right) are shown by pie charts. Compositions of matcha and green tea extract varied little by harvest year. # The denominator is the amount of total polyphenols.*The values (µg) shown are estimated total polyphenol amounts in 1.5 g of matcha powder, which is equivalent to the weight mixed with hot water (70 mL) to make a cup of Matcha (100 mL) in Japanese traditional tea ceremony. **The values (µg) shown are estimated total polyphenol amounts in 0.5 g of dried powder of green tea extracts, which is equivalent to the contents in a cup of green tea (150 mL).Data shown are the copy number of universal 16S rRNA gene in saliva of 45 individuals before (open bars) and after (red bars) intervention.

**Supplemental Figure 5: The copy number of 16S rRNA gene in saliva samples of patients.**

Data shown are the copy number of the universal 16S rRNA gene in saliva of 45 individuals before (open bars) and after (red bars) intervention.

**Supplemental Figure 6: The amounts of six periodontopathic bacterial species in saliva sample of each patient.**

The numbers of the following six bacterial species in saliva were quantified by real-time PCR: *P. gingivalis* (Pg), *T. forsythia* (Tf), *T. denticola* (Td), *F, nucleatum* (Fn), *P. intermedia* (Pi), and *A. actinomycetemcomitans* (Aa). Data are shown as radar charts of with dimensions composed of the six species. The blue and red lines denote the results before and after intervention, respectively. Data shown are the copy number of the universal 16S rRNA gene in saliva of 45 individuals before (open bars) and after (red bars) intervention.

**Legends of Supplemental Movies**

**Supplemental Movie 1: Real-time observation of cells after treatment with ME at 1 mg/mL.**

HS-AFM observation of *P. gingivalis* was monitored with a BIXAM system (Olympus). *P. gingivalis* cells were immobilized on glass slides and treated with 1 mg/mL ME. Images were taken at 0.1 fps. The constructed movie is shown at 70 times the original speed. The area of each image is 1980 x 1485 nm^2^ (*x* x *y*).

**Supplemental Movie 2: Real-time observation of cells after treatment with ME at 0.1 mg/mL.**HS-AFM observation of *P. gingivalis* was monitored with a BIXAM system (Olympus). *P. gingivalis* cells were immobilized on glass slides and treated with 1 mg/mL ME. Images were taken at 0.1 fps. The constructed movie is shown at 70 times the original speed. The area of each image is 4020 x 3015 nm^2^ (*x x* *y*).
